# Intraspecies sequence-graph analysis of the *Phytophthora theobromicola* genome reveals a dynamic structure and variable effector repertoires

**DOI:** 10.1093/g3journal/jkaf256

**Published:** 2025-10-27

**Authors:** Jadran F García, Rosa Figueroa-Balderas, Alina S Puig, Indrani Kakati, Michael E H Matson, Shahin S Ali, Bryan A Bailey, Jean-Philippe Marelli, Dario Cantu

**Affiliations:** Department of Viticulture and Enology, University of California Davis, Davis, CA 95616, United States; Department of Viticulture and Enology, University of California Davis, Davis, CA 95616, United States; Foreign Disease - Weed Science Research Unit, USDA-ARS, Frederick, MD 21702, United States; Sustainable Perennial Crops Laboratory, USDA-ARS, Beltsville, MD 20705, United States; Plant Science Center, Mars Wrigley, Davis, CA 95616, United States; Sustainable Perennial Crops Laboratory, USDA-ARS, Beltsville, MD 20705, United States; Sustainable Perennial Crops Laboratory, USDA-ARS, Beltsville, MD 20705, United States; Plant Science Center, Mars Wrigley, Davis, CA 95616, United States; Department of Viticulture and Enology, University of California Davis, Davis, CA 95616, United States; Genome Center, University of California Davis, Davis, CA 95616, United States

**Keywords:** *Phytophthora theobromicola*, cacao pathogens, sequence graph, RxLR effectors, transposable elements, genome plasticity, host adaptation, genome assembly

## Abstract

*Phytophthora theobromicola* is an emerging cacao pathogen recently identified in Brazil as an aggressive agent of black pod rot. We generated genome assemblies for two *P. theobromicola* isolates using long-read sequencing and five additional isolates using short reads. Comparative analysis revealed a genome size and predicted gene content comparable to *P. citrophthora*, a closely related species with a broad host range that includes both citrus and cacao. An intraspecies sequence-graph analysis revealed a highly dynamic genome structure with high proportion of variable effectors. Syntenic orthology analysis across 13 *Phytophthora* species identified orthologous gene groups conserved only in cacao pathogens and others specific to *P. theobromicola*. RxLR effectors and CAZymes were particularly enriched among lineage-specific syntenic groups, with RxLRs preferentially located near transposable elements and within gene-sparse, repeat-rich regions. Transcriptome analysis of infected cacao tissues showed that 88% of predicted effectors were expressed, with pods exhibiting the highest number of upregulated genes. Notably, several RxLRs classified as *P. theobromicola-*specific syntenic orthologs were highly expressed in infected tissues, suggesting that these lineage-specific effectors may play key roles in host-pathogen interactions unique to cacao. Together, our findings highlight the dynamic architecture and functional plasticity of the *P. theobromicola* genome, providing foundational insights into its virulence strategies and supporting future studies on host adaptation and effector evolution in emerging cacao pathogens.

## Introduction

The worldwide chocolate industry, valued at over 130 billion US Dollars annually (Zion Market Research 2023) faces significant threats from multiple cacao diseases that reduce both yield and quality (Marelli et al. 2019). It is estimated that up to 38% of global annual cacao bean production is lost to disease, with more than half of these losses attributed to *Phytophthora* species (Marelli et al. 2019). Members of this genus can infect all parts of the cocoa plant, causing black pod rot, stem canker, and leaf blight (Firman and Vernon 1970; Appiah et al. 2004; Marelli et al. 2019). Among the major cacao diseases, black pod disease (also known as black pod rot) is particularly devastating, causing yield losses of 20–30% on average, with some farmers in wetter regions reporting complete crop failure (Drenth and Guest 2013). The lesions caused by the disease can appear at any stage of development and at any part of the pod, starting as small, hard, dark spots and growing rapidly to cover the entire pod damaging the beans inside (Guest 2007). The progression of the disease is influenced by multiple factors, including the *Phytophthora* species, cacao genotype, humidity, rainfall, and temperature (de Oliveira 2005; Guest 2007; Puig et al. 2018).

The development of disease is facilitated by an arsenal of proteins secreted by the pathogen that interact with host cells (Kamoun 2006; McGowan and Fitzpatrick 2017). These proteins, collectively referred to as effectors, are broadly classified by their site of action: apoplastic effectors act in the extracellular space, while cytoplasmic effectors are translocated into the host cell. Apoplastic effectors include toxins such as necrosis- and ethylene-inducing peptide 1-like proteins (NLPs), which have been shown to trigger cell death in model plants. In *Phytophthora* species, NLPs are associated with disruption of host membrane integrity (Lenarčič et al. 2017; Wang and Wang 2018). The apoplastic compartment also contains a diverse set of hydrolytic enzymes that degrade plant cell wall components, facilitating nutrient acquisition. Among these, carbohydrate-active enzymes (CAZymes)—including cutinases, pectinases, and glycoside hydrolases are enriched in *Phytophthora* genomes (Jiang and Tyler 2012; Ma et al. 2015; Wang and Wang 2018). Among cytoplasmic effectors, two major families have been well characterized in *Phytophthora*: RxLR and Crinkler (CRN) effectors (Whisson et al. 2007; Schornack et al. 2010; McGowan and Fitzpatrick 2017). RxLR effectors are defined by an N-terminal motif containing “RxLR” and “EER” sequences, which are necessary for host cell translocation (Jiang and Tyler 2012; McGowan and Fitzpatrick 2017; Wang and Wang 2018). In some cases, this translocation can occur independently of additional pathogen-encoded machinery (Dou et al. 2008; Wang et al. 2023). Functionally, RxLR effectors have been shown to either suppress host programmed cell death or, conversely, induce it, depending on the host context and specific effector (Wang et al. 2011). Crinklers (CRNs) are characterized by an “LxLFLAK” motif that facilitates translocation (Schornack et al. 2010; McGowan and Fitzpatrick 2017) and are known to localize to the host nucleus (Schornack et al. 2010; Stam et al. 2013). CRNs have been implicated in both induction and suppression of host cell death (Liu et al. 2011; Stam et al. 2013), although the specific mechanisms are not yet fully understood. Both RxLRs and CRNs are often located in gene-sparse, repeat-rich regions of the genome, which are associated with accelerated evolution and diversification of virulence factors (Stassen and Van den Ackerveken 2011).

At least five *Phytophthora* species have been reported to cause black pod rot with significant commercial impact: *P. palmivora, P. megakarya*, *P. capsici*, *P. citrophthora*, and, more recently, *P. theobromicola* (Surujdeo-Maharaj et al. 2016; Marelli et al. 2019; Decloquement et al. 2021). Both *P. palmivora* and *P. megakarya* have undergone recent and independent whole-genome duplications, with *P. megakarya* possessing one of the largest known *Phytophthora* genomes at approximately 222 Mbp (Morales-Cruz et al. 2020). This duplication contributed to a substantial increase in gene content, with an average of 57,577 protein-coding genes in *P. megakarya* and 36,778 in *P. palmivora*. These expansions include a high number of putative virulence factors, particularly effector proteins such as RxLRs, CRNs, and NPP1-like proteins (a class of NLP), underscoring the role of gene duplication and structural variation in effector diversification and pathogen adaptation.

*Phytophthora theobromicola* sp. nov was recently isolated from a set of isolates obtained from cacao pods exhibiting symptoms of black pod disease (Decloquement et al. 2021). The identification of the new species was based on a combination of morphological features and multilocus phylogenetic analysis. Molecular markers used for this analysis included sequences from the β-tubulin, elongation factor 1-alpha, heat shock protein 90, internal transcribed spacer (ITS), and cytochrome c oxidase subunits I and II genes, which together supported the designation of *P. theobromicola* as a distinct species. *Phytophthora theobromicola* isolates were found to be more aggressive than *P. palmivora*, suggesting a high virulence potential (Decloquement et al. 2021).

In response to its recent emergence and demonstrated pathogenicity, we present here the first genomic resources for *P. theobromicola*, providing a foundation for future studies on its evolutionary history, adaptive potential, and virulence mechanisms. We assembled high-quality diploid genomes for two isolates using long-read sequencing and generated short-read assemblies for five additional isolates. We performed functional annotation of candidate effectors and constructed a pangenome graph including all isolates to assess intraspecific variability in effector content. Comparative genomic analysis with multiple *Phytophthora* species revealed groups of syntenic orthologs shared among cacao-infecting species, as well as orthologs unique to *P. theobromicola*. To characterize gene expression dynamics during infection, we analyzed RNA-seq data from inoculated cacao tissues, revealing the large set of predicted effectors expressed during pod infection.

## Materials and methods

### Inoculation and nucleic acid extraction

All inoculations were performed with isolate P0449, obtained from ATCC and originally collected from diseased cacao in Bahia, Brazil. All plant material used for inoculations was grown at the USDA-ARS Research Station in Miami, FL. Clones G-026 and T-484 were selected based on the availability of sufficient material at the appropriate physiological stage.

First, the stems of six-week-old open-pollinated seedlings of the G-026 (bioreps *n* = 10) were pierced with a 1 mm diameter probe, to a depth of 2 mm. Then 3 mm discs of 20% v8 agar with actively growing mycelia of *P. theobromicola* (P0449) were placed mycelial-side down over the wound of five of the seedlings. Similarly, 3 mm discs of 20% v8 agar without mycelial growth were placed over the wounds of other five seedlings to serve as negative control. Plugs were covered with damp 1 cm^2^ filter paper, and wrapped in parafilm, as described in (Puig et al. 2021). Seedlings were placed in laboratory growth chambers at 25 °C with 12 h light and 12 h darkness. Stems were harvested five days post inoculation, flash frozen in liquid nitrogen, then lyophilized for RNA extractions.

Expanded, tender leaves from clone T-484 were divided in half, lengthwise along the midvein, with one half inoculated with 20% V8 agar media colonized with *P. theobromicola* (P0449) (bioreps *n* = 6), and the other half inoculated with uncolonized plugs of 20% V8 agar media (bioreps *n* = 6). All inoculations were done on the abaxial side of the leaves, between minor veins then placed at 25 °C in the dark (Schmidt et al. 2023). Approximately 2 to 4 plugs were placed on each leaf, depending on the size. The plugs were removed 48 h post inoculation then leaves were flash frozen in liquid nitrogen and lyophilized for RNA extractions.

Pod inoculations were done using 2.5 × 3 cm pieces of unripe cacao pods from clone T-484. A 6-mm-diameter cork borer (2 mm depth) and the excised tissue was replaced with 6 mm discs of 20% v8 agar with actively growing mycelia of *P. theobromicola* (P0449). Inoculation sites were covered with a damp 1 cm^2^ filter paper, and wrapped in parafilm to prevent desiccation. Five pod pieces (bioreps *n* = 5) were inoculated with *P. theobromicola* and five (bioreps *n* = 5) were inoculated with uncolonized 20% v8 agar. Pod pieces were placed in sealed plastic bags and incubated in a growth chamber at 25 °C in the dark. After 48 h, pod pieces were flash frozen in liquid nitrogen and lyophilized for RNA extractions.

The total RNA from the inoculated, control tissues and from the pure mycelia of the P0449 isolate was extracted as previously described in Morales-Cruz et al. (2020). Only the bioreps samples with good quality RNA were sequenced including 3 inoculated leaves, 3 control leaves, 4 inoculated pods, 5 control pods, 3 inoculated stems, 3 control stems, and 3 mycelial samples from in vitro culture. Likewise, the rest of the *Phytophthora theobromicola* isolates used for this study were obtained from symptomatic pods of different cultivation sites in Bahia, Brazil (Decloquement et al. 2021). The genomic DNA (gDNA) for short-read sequencing and the high molecular weight (HMW) gDNA for SMRT sequencing was also extracted as previously described in Morales-Cruz et al. (2020).

### Sequencing library preparation

The high molecular weight (HMW) gDNA of *Phytophthora theobromicola* P0449 and MB01960 was fragmented using a 26G blunt needle (SAI Infusion Technologies, IL, USA) by aspirating the entire volume and passing the sample through the needle fifteen times. After shearing, the sample was cleaned and concentrated using 0.45× AMPure PB beads. The size distribution of the sheared gDNA fragments was evaluated using pulsed-field gel electrophoresis (Pippin Pulse, Sage Science, MA, USA) prior to library preparation. Continuous Long Read (CLR) libraries were prepared using the SMRTbell Express Template Prep Kit 2.0 (Pacific Biosciences, CA, USA) following the manufacturer's instructions. Up to 5 µg of SMRTbell template was size-selected with the Sage Blue Pippin (Sage Science, MA, USA) using a cutoff range of 17–80 kb. The size-selected library was cleaned with 1× AMPure PB beads and sequenced on the PacBio Sequel II platform (DNA Technology Core Facility, University of California, Davis). Likewise, The genomic DNA of the remaining *P. theobromicola* isolates was sheared to approximately 450 bp using a Covaris E220 sonicator (PerkinElmer, MA, USA). DNA-seq libraries were prepared from the sheared DNA using the KAPA LTP Library Preparation Kit (Roche Diagnostics, IN, USA). The libraries were sequenced in 150 bp paired-end mode (2 ×150) on an Illumina HiSeq 4000 (Novogene Co. Ltd., Beijing, China).

The RNA extracted from the pure mycelium, inoculated tissue and control tissue was used to prepare the RNA-seq libraries. A total of 500 ng of RNA per sample was used as input to prepare the libraries using the TruSeq RNA Sample Preparation Kit v2 (Illumina, CA, USA), following the manufacturers' protocols with individual barcoding. Final libraries were assessed for quantity and quality using a High Sensitivity chip on a Bioanalyzer 2100 (Agilent Technologies, CA, USA) and a Qubit fluorometer (Life Technologies, CA, USA). The RNA-seq libraries were sequenced in 150 bp paired-end mode (2 ×150) by IDSeq on an Illumina NovaSeq 6000.

### Genome assembly

The assembly of *P. theobromicola* isolates MB01960 and P0449 were assembled using SMRT reads with FALCON-Unzip ver. 2017.06.28-18.01 (Chin et al. 2016) using a custom pipeline published in (Minio et al. 2019) and available at https://github.com/andreaminio/FalconUnzip-DClab. Different parameter combinations were tested to obtain the least fragmented assembly as described in (Morales-Cruz et al. 2020). Haplotype phasing was performed with default parameters (Chin et al. 2016) to obtain the primary contigs and the haplotigs. Primary contigs and haplotigs were polished with Arrow (from ConsensusCore2 v.3.0.0) using raw long reads. Primary contigs and haplotigs were scaffolded using SSPACE-Longreads v.1.1 (Boetzer and Pirovano 2014). Merqury v.1.3 (Rhie et al. 2020) was used on diploid mode to evaluate the genome consensus quality (QV), completeness and haplotype duplication rate using a 21-mer approach from paired-end short reads of the same isolates. Additionally the same paired-end short reads were mapped to the primary assembly using pbrun fq2bam v4.5.0-1 (Zhu et al. 2025) and the homozygous SNPs on non-repeats regions called by pbrun haplotypecaller v.4.5.0-1 (Zhu et al. 2025) were used as direct approximation of the error rate and QV of the genomes.

The genomes of the isolates in Supplementary table 1 were assembled using paired-end short read sequencing data. Raw reads were quality-filtered and adapter clipped using Trimmomatic v.0.36 (Bolger et al. 2014), with the following settings: ILLUMINACLIP:2:30:10 LEADING:7 TRAILING:7 SLIDINGWINDOW:10:20 MINLEN:36. SPAdes v4.0.0 (Prjibelski et al. 2020) was used to assemble the quality-filtered reads with the careful option and automatic read coverage cutoff after optimizing the multiple kmer combination (-k 55,77,99,111,127). These contigs were labeled “main” if their length was at least 1,000 bp and “short” for contigs smaller than 1,000 bp. To assess the assembly completeness of the genomes, we performed Benchmarking Universal Single-Copy Orthologs (BUSCO v.5.4.2; (Manni et al. 2021) analysis with the stramenopiles_odb10 lineage dataset.

### Repeat and gene annotation

The concatenated genomes of all *Phytophthora* species in Table 1 were used to predict repeat models using RepeatModeler v. 2.0.5 (Smit et al. 2015; Flynn et al. 2020) with default parameters and -LTRStruct option to predict LTR retrotransposons based on their structure. The predicted models were concatenated with the RepeatMasker-RepBase database (release 20181026) and used with RepeatMasker v.4.1.5 (Smit et al. 2025) to mask the repeats in each *P. theobromicola* genome. The tool maskFastaFromBed (Quinlan 2014) was used to softmask the repeats in all the genomes.

**Table 1.** Genome assembly and gene annotation statistic of *P. theobromicola* primary assembly and other Phytophthora species affecting Theobroma cacao.

Species	*P. theobromicola* (MB01960)	*P. theobromicola* (P0449/ATCC)	*P. megakarya* (PmGH34)	*P. palmivora* (PpGH49)	*P. capsici* (LT1534)	*P. citrophthora* (STE-U-9442)
Haploid assembly size (Mb)	47.5	45.6	214.2	115.5	94.2	48.5
Coverage	757×	767×	>150×	>150×	35×	521×
No. of scaffolds	52	27	502	240	782	155
N50 (Mb)	1.9	2.1	0.8	0.9	0.5	0.9
L50 (scaffold no.)	11	7	78	43	44	16
Complete BUSCOs in assembly (%)	100	96	100	100	100	100
Complete and single copy BUSCOs (%)	97	96	63	57	78	100
Complete and dup. BUSCOs (%)	3	0	37	43	22	0
Fragmented BUSCOs (%)	0	0	0	0	0	0
Missing BUSCO (%)	0	4	0	0	0	0
Repeats masked (Mb)	13.7	12.8	139	56.6	46.4	14.4
Repeats masked (%)	28.8	28.1	64.9	49	49.3	29.6
No. of CDS	16,387	15,771	53,476	31,821	23,748	16,447
Mean protein size	471	469	340	427	434	498
BUSCOs in gene prediction (%)	97	95	95	95	99	91
Mean gene density (gene/10 kb)	4.0	4.0	2.8	3.1	2.8	3.9
Source	This work	This work	Morales-Cruz et al. 2020	Morales-Cruz et al. 2020	(Stajich et al. 2021)	(Möller et al. 2024)

The RNAseq data obtained from pure mycelium and inoculated material was quality filtered and mapped to all softmasked *P. theobromicola* genomes individually using Hisat2 v.2.2.1 (Kim et al. 2019) with –dta –very-sensitive options. The gene prediction *P. theobromicola* genomes was made with Braker2 v2.1.6 (Lomsadze et al. 2005, 2014; Stanke et al. 2006, 2008; Gotoh 2008; Ter-Hovhannisyan et al. 2008; Li et al. 2009; Barnett et al. 2011; Iwata and Gotoh 2012; Buchfink et al. 2015; Hoff et al. 2016, 2019; Brůna et al. 2020, 2021) using as evidence the mapped RNAseq reads (bam file) and a custom protein database using as base OrthoDB v.11 (Kuznetsov et al. 2023) and predicted proteins of *P. megakarya*, *P. palmivora*, *P. citrophthora* and *P. capsici*. This annotation was cleaned to remove proteins with internal stop codons and without stop codons.

To enhance gene model consistency across all the assemblies and mitigate the effects of fragmentation of some genomes, we performed gene porting by projecting a curated, non-redundant gene set derived from *P. theobromicola* MB1960 on all genomes. The set of non-redundant genes were obtained using the coding sequences of *P. theobromicola* MB1960 with Orthofinder 2.5.5 (Emms and Kelly 2019) and filtering each orthogroups to obtain the longest representative. This non-redundant set was used to create artificial reference genome and gff3 from the original *P. theobromicola* MB1960. The artificial reference was used with LiftOn v1.0.5 (Shumate and Salzberg 2021; Chao et al. 2024) and each *P. theobromicola* assemblies with the options “-a 0.9 -s 0.9 -exclude_partial -copies -sc 0.95 -polish -cds”. The new lifted annotation was cleaned to remove proteins with internal stop codons (identified with the character *) and proteins without stop codon. The lifted annotation was intersected against the initial braker annotation and the braker genes with no overlap with the lifted genes were kept in the final annotation of each genome. BUSCO v.5.4.2 (Manni et al. 2021) with the stramenopiles_odb10 lineage dataset and the protein mode was used to asses completeness.

### Functional and effector annotation

The functional annotation was made focusing on effectors and therefore the first task was to identify the secretome. SignalP3 (Dyrløv Bendtsen et al. 2004) was selected for this task over newer versions because previous studies have found that it is the most sensitive at detecting oomycete signal peptides (Sperschneider et al. 2015; McGowan and Fitzpatrick 2017). The mature proteins were used as input for TMHMM v2.0 (Krogh et al. 2001). Proteins were classified as secreted if they had a signal peptide and no transmembrane predicted by TMHMM. Then all the secreted proteins were checked with EffectorO (Nur et al. 2023) and its machine learning models to predict potential effectors. Next, all the proteins were annotated with the InterProScan v.5.69 pipeline and the databases Pfam, PANTHER, SUPERFAMILY and NCBIfam. To annotate CAZymes, dbCAN3 (Zheng et al. 2023) with the options “–dia_eval 1e-102 –hmm_eval 1e-15 –hmm_cov 0.35” was run locally on all proteins. Annotations validated by two of three tools in dbCAN3 were kept as CAZymes. The R package effectR v.1.0.2 (Tabima and Grünwald 2019) was used to annotate RxLR and CRN in all the genomes. First the “regex.search” was used to obtain a training list of RxLR and CRN containing the well-known domains, then, a hmm model was trained based on these lists and the proteins were compared against the model to obtain a non-redundant set of putative RxLR and CRN effectors. Additionally, CRN effectors were also annotated using the hmm models created by Morales-Cruz et al., (2020). Each of the models were used to search all the predicted proteins with a e-value of 0.01 using HMMER v3.2.1 (Wheeler and Eddy 2013). Next, if the protein was classified as secreted and had a functional annotation (in any of the databases with any of the annotation methods used) consistent with known effectors (based on McGowan and Fitzpatrick 2017) they were considered putative effectors. All CRN-annotated proteins, regardless of their secretion signal annotation, were also presented as putative effectors. Supplementary table 2 presents the functional annotation with all the methods. Supplementary table 3 contains the secretome with the putative effector proteins corresponding functional annotation used in the rest of the analyses.

### Sequence graph of effector variability

The genomes of the seven isolates of *P. theobromicola* (including haplotigs) were used to build the sequence graph with the Nextflow v.24.10.2 pipeline nf-core/pangenome (v1.1.2-g0e8a387; https://github.com/nf-core/pangenome/tree/1.1.2; 10.5281/zenodo.10869589; Heumos et al. 2024). The defaults parameters were used except the “wfmash_segment_length” that was set to 3000 based on a previous optimization. The resulting sequences graph was used to classify the nodes in 3 categories. The core nodes were those present in all the isolates genomes, the dispensable nodes were those in more than one isolate but not in all of them, and the private nodes were those present exclusively in a single isolate. The total set of nodes is considered the full repertoire. Gene classification was based on node composition. Each gene was assigned to the category representing the majority of its length. For example, if a gene consisted of 33% core nodes, 39% dispensable nodes, and 28% private nodes, it was classified as dispensable. No ambiguous cases were found in any isolate. Models for sequences, genes, secreted proteins, and effectors were generated through iterative combinations of different isolates in the sequence graph. Since we decided to include the primary and haplotigs of both long-read assembled genomes, the nodes present in the isolate (regardless of presence in primary, haplotigs or both) were counted only once to avoid over-counting. Similarly, for the gene models, we took the genes in the primary assembly and additional genes from the haplotigs that had an overlap below 50% with genes from the primary assembly. This approach increased only by 4 and 5% the total number of genes of the 2 long-read primary assemblies while allowing us to also include the within isolate variability. Finally nodes and the genes were reclassified at each combination of genomes and the results were plotted as boxplots to include the variation between different genome combinations. To visualize the trends per category smoothed lines were applied with a smoothing span of 0.8 and 95% confidence intervals.

### Syntenic orthology inference

The primary genes of *P. theobromicola* MB01960 and P0449 plus the genes of the 9 other *Phytophthora* species (Supplementary table 4) with different hosts, including cacao, were used to perform the syntenic orthology analysis. The CDS and bed files of these species were processed with GENESPACE v.1.3.1 (Lovell et al. 2022) using default parameters. GENESPACE incorporates protein similarity into syntenic blocks detection with MCScanX v.1.0.0 (Wang et al. 2012) and employs Orthofinder v2.5.5 (Emms and Kelly 2019) to identify orthologs and paralogs within these synteny-constrained regions. After running the main pipeline the function “syntenic_pangenes” was applied iteratively using each genome as reference. The genes with a flag PASS were count in each genome to obtain binary matrix with the reference gene and presence absence in all the other genomes. Genes with the flag PASS in at least one other genome and not with itself was counted as syntenic ortholog (SynOr). A gene was classified as SynOr conserved in all *Phytophthora* species if they were present in all the genomes (including at least one *P. theobromicola* isolate). A gene was classified as SynOr conserved only in cacao pathogens if they were present in all cacao pathogens (*P. theobromicola, P megakarya, P. palmivora, P. capsici* and *P. citrophthora* based on published reports) and no more than three other species. And a gene was considered a SynOr conserved only in *P. theobromicola* in they are only present in the *P. theobromicola* genomes. The effector annotation was intersected with this information to obtain the functions of the effectors in these SynOr categories. The phylogenetic tree obtained from Orthofinder was used to account for the phylogenetic relatedness of the species in this analysis.

### RxLR homology network analysis

The total set of putative RxLR effectors in the cacao pathogens were compared against each other using the blastp of Diamond v2.1.9.163 (Buchfink et al. 2021) with the options “–evalue 1e-10 –max-target-seqs 0 –outfmt 6 qseqid sseqid pident length evalue bitscore”. The result file was filtered to keep only matches with at least 30% of coverage and at least 50% identity. The network was visualized with Gephi v.0.1.0 (Bastian et al. 2009) using the bitscore as edges. The network was arranged using the Fruchterman-Reingold layout (Fruchterman and Reingold 1991) and the cluster were defined by the modularity class calculated within Gephi. The color annotation was done using the SynOr classification. The homologous proteins of each clusters with SynOr conserved only in *P. theobromicola* were aligned with Muscle v.5.3 (Edgar 2022) and Maximum Likelihood (ML) trees were created for each cluster using RAxML-NG v.0.9.0 (Kozlov et al. 2019) with the options “–tree pars{10} –bs-trees 100 –model WAG + I + G4”. The trees were visualized and annotated with the R packages Ape v.5.8-1 (Paradis and Schliep 2019), phytools v.2.4-4 (Revell 2024) and ggtree v.3.14.0 (Xu et al. 2022).

### Gene duplication classification

The proteins sequences of each *P. theobromicola* primary assembly were blasted against each other using Diamond v2.1.9.163 with the options “evalue 1e-3 –outfmt 6”. Matches with at least 50% coverage and identity were kept. These results passed through the MCScanX v.1.0.0 pipeline (Wang et al. 2012) and the program “duplicate_gene_classifier” distributed with MCScanX was used to classify the observed duplication as segmental (match genes in syntenic blocks), tandem (continuous repeat), proximal (close region but not adjacent), dispersed (none of the previous categories) and singleton (no duplication). This classification was intersected with the SynOr classification and the effector annotation to extract further insights.

### Transposable element proximity and intergenic space

The gene coordinates of the primary assemblies *P. theobromicola* MB01960 and P0449 were concatenated and sorted. Similarly, the Class I and Class II TE coordinates of both genomes were concatenated and sorted (one file per class type). The tool “closest” within BEDtools v2.29.1 (Quinlan 2014) with the options “-d -io” was used to determine the distance of each gene to the closest TE on each class. Then, for each gene, the closest TE (of any kind) was selected, and the distance was recorded. Next, the genes were grouped per effector functional annotation, and a Kolmogorov–Smirnov test was used to compare the distributions of TE proximity between gene groups. A significant result indicates that one group is consistently closer or further from TEs than the other group. This information was intersected with the gene duplication classification and effector annotation to gain more insights.

The intergenic space was calculated using the tool “closest” within BEDtools v2.29.1 with the coordinates of all the genes and the options “-D a -io -k 2”. The distance to the closest gene was recorded on both sides of the genes when possible. Genes with only one closest gene were located at the end of the scaffolds. This information was intersected with RxLR effector annotation, proteins with secretion signal and SynOr categories to gain further insights.

### Expression analysis

RNAseq paired-end reads of all samples (mycelium, inoculated tissues and control tissues) trimmed with Trimmomatic v0.36 (Bolger et al. 2014) with the options “LEADING:7 TRAILING:7 SLIDINGWINDOW:4:15 MINLEN:50”. The genome of *Theobroma cacao* (GCF_000208745.1; (Argout et al. 2017) was concatenated with the primary assembly of *P. theobromicola* P0449 to use as reference for mapping. The reads were mapped using the Nextflow v.24.10.2 pipeline nf-core/rnaseq (v3.18.0-gb96a753; https://github.com/nf-core/rnaseq/; 10.5281/zenodo.1400710) with the options “pseudo_aligner: salmon, extra_salmon_quant_args: –seqBias –gcBias”. The transcripts and gff files were also a concatenation of both cacao and pathogen. Estimation and statistical analysis of expression level using the salmon_merged_gene_counts data from salmon were performed using the DESeq2 v.1.46.0 (Love et al. 2014) in R. The normalized counts in inoculated tissue and control tissue were calculated within DESeq2 using the size factors account for differences in library depth (Supplementary table 5). The differential expressions (DE) analysis between infected tissues was calculated with the default parameters of DESeq. The contrasts of each pair of tissues were extracted and the thresholds for DE were set to Log2FC larger than 1 and adjusted *P*-value lower than 0.05. Upregulated genes on each tissue were extracted from the contrast with the other 2 tissues to create an upset plot. The Supplementary table 6 present the upregulated genes and their classification per tissue type. Last, the DE genes between in-planta (inoculated tissue) and in-vitro (mycelium) were calculated using only the pathogen transcripts in the inoculated dataset. The datasets were processed with the default parameters of DESeq including the size factors normalization to account for differences in library depth. The DE genes were selected based on the same thresholds as before. The in-planta upregulated genes were binned into windows of Log2FC of five units (from 1 to 5, from 5 to 10, from 10 to 15 and so on) for figure preparation. The full table of DE genes between in-planta vs in-vitro is presented in Supplementary Table 7.

## Results

### Genome assembly of *Phytophthora theobromicola*

The genomes of two *P. theobromicola* isolates, MB01960 and P0449, collected in Bahia, Brazil, were assembled using PacBio CLR long-read sequencing (Table 1). Each assembly included both primary contigs and associated haplotigs, which were uniformly distributed across the genome, consistent with a diploid representation. The haploid genome sizes were estimated at 47.5 Mbp for MB01960 and 45.6 Mbp for P0449, with sequencing coverage exceeding 700×. The QV of the genomes calculated by the kmer approach was 38.5 and 43.1 for the isolates MB01960 and P0449, respectively, while the completeness was approximated 97 and 95%. Similarly, the QV calculated by homozygous SNPs (error rate) was 50.1 and 54.7 for the two isolates, respectively.

The genome sizes place *P. theobromicola* among the smaller cacao-infecting *Phytophthora* species, similar to *P. citrophthora*, and considerably smaller than *P. capsici* and *P. palmivora* (approximately half), and *P. megakarya* (less than one-quarter). Genome completeness, assessed using BUSCO on both genomic sequences and predicted proteomes, was high for both isolates, exceeding 96%. Additionally, the low or absent complete duplicated BUSCOs in *P. theobromicola* isolates (Table 1) is consistent with the haplotype duplication calculated with merqury spectra-cn.hist, which show duplication rate of 0.14 and 0.15% for the isolates MB01960 and P0449, respectively. Predicted gene counts followed a pattern consistent with genome size, showing similar values to those observed in *P. citrophthora*. In addition, five more *P. theobromicola* isolates were sequenced using short-read technologies and *de novo* assembled (Supplementary Table 1). These assemblies had an average size of 43.8 ± 0.3 Mbp (considering scaffolds >1 kb), with coverage above 400×. The average genome completeness for these assemblies was 88.8 ± 2.0%, and the number of predicted genes was approximately 32% lower than in the long-read assemblies, likely reflecting limitations in assembly contiguity and completeness using short-read sequencing.

### Effector identification and functional annotation

*Phytophthora* species, like other oomycete pathogens, are known to secrete a large repertoire of effector proteins that facilitate host colonization and suppress plant defenses (Wawra et al. 2012; McGowan and Fitzpatrick 2017). We functionally annotated the putative effector repertoires of the five cacao-infecting *Phytophthora* species listed in Table 1, including both *P. theobromicola* isolates. To identify secreted proteins, we first predicted the secretome of each species using SignalP3 in combination with transmembrane domain prediction, retaining only proteins with a secretion signal and no transmembrane domains outside the signal peptide. Proteins with predicted secretion signals and domain annotations consistent with known effector categories were classified as putative secreted effectors. From a total of 157,650 predicted proteins across the primary assemblies of all species, 10,931 were identified as putatively secreted. On average, 49.5 ± 1.8% of the secretome per species was annotated as putative secreted effectors (Table 2). In *P. theobromicola*, RxLR effectors and CAZymes together accounted for more than 66% of the predicted effectors, while in *P. megakarya*, these two categories comprised over 76%, with RxLRs alone representing 55.9% (Table 2). Among the CAZymes, we identified numerous enzymes implicated in cell wall degradation, including those targeting pectin, lignin, and hemicellulose. Additionally, several elicitins, NLPs, and phytotoxins were detected in the secretome of all species. A full list of annotated effector genes, including those from the short-read assemblies, is provided in Supplementary Tables 2 and 3. Summary statistics by effector category are available in Supplementary Table 8.

**Table 2.** Counts of effector proteins classified per category or function in the species in study.

Category	*P. theobromicola* (MB01960)	*P. theobromicola* (P0449/ATCC)	*P. megakarya* (PmGH34)	*P. palmivora* (PpGH49)	*P. capsici* (LT1534)	*P. citrophthora* (STE-U-9442)
Number of secreted proteins	1424	1347	3170	2315	1653	1022
Number of secreted effectors	661	607	1806	1220	786	495
Secreted effector (%)	46.4	45.1	57	52.7	47.5	48.4
RxLR	208	196	1009	569	282	203
CRN^a^	101(3)	88(2)	157(0)	159(0)	199(3)	215(4)
CAZymes	232	205	368	340	294	149
Glycoside hydrolases	107	107	182	187	132	70
Cellulases	12	11	19	18	14	7
Chitinases	2	2	2	3	3	5
Cutinases	6	6	11	7	9	4
Hemicellulose degradation	25	25	51	52	32	17
Lignin degradation	10	9	13	6	8	4
LPMOs	65	47	87	91	73	25
Pectin degradation	66	63	113	94	81	59
Elicitins	46	41	45	48	34	38
NLP	57	47	146	113	43	28
Phytotoxins	5	5	0	1	5	6
Trypsin & Trypsin like proteins	18	17	56	24	23	12
Kazal-type Protease inhibitors	18	17	72	37	23	15
Papain and papain like family cysteine proteases	7	7	8	8	12	4
Small cysteine-rich proteins	29	29	57	35	26	15

The number in parenthesis represents the Crinkler effectors with secretion signal.

^a^Total number of annotated Crinkler effectors.

### Sequence graph analysis reveals high intraspecific variability of the effector repertoire in *P. theobromicola*

**Fig. 1.**
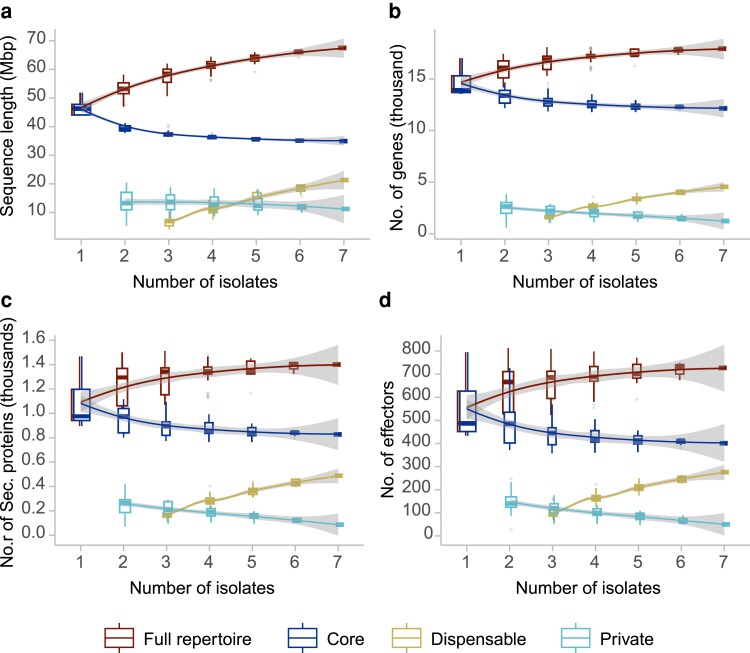
Variability within the *P. theobromicola* species represented as sequence graph models of a) sequence length, b) number of total genes, c) number of secreted proteins, and d) number of effectors. All panels share the same color legend.

Genomic variability among isolates of a single species has been shown to significantly impact the composition and diversity of virulence factors in plant pathogens, contributing to their ability to adapt to diverse environmental conditions (Garcia et al. 2024). Analyses of *P. palmivora* and *P. megakarya* revealed that effector genes particularly those belonging to the RxLR family are overrepresented among genes under positive selection (Morales-Cruz et al. 2020). To assess intraspecific effector variability in *P. theobromicola*, we constructed a sequence graph that integrates the genomic content of all available isolates. This approach enables the identification of both conserved and variable regions across genomes and allows for classification of effector genes into three categories: core (present in all isolates), dispensable (shared by more than one but not all), and private (unique to a single isolate). The graph was constructed using primary contigs and haplotigs from the two long-read assemblies, along with the five short-read genome assemblies, resulting in a combined graph spanning 67.5 Mbp (Fig. 1a). This represents an 83% compression relative to the ∼400 Mbp cumulative length of the individual assemblies, reflecting a high degree of shared sequence content and capturing structural variation across isolates.

After classifying the genes of each isolate based on their position in the graph (core, dispensable, or private), we observed a linear increase in the number of dispensable genes with each additional genome included (Fig. 1b). A similar pattern was observed when focusing specifically on secreted proteins and effector genes (Fig. 1, c and d): the number of dispensable effectors increased linearly across genomes, while the total effector repertoire appeared to begin plateauing after the third isolate was added. A detailed analysis of the final effector graph revealed that, on average, 402 ± 42 effector genes per genome were classified as core, representing 84.0 ± 0.7% of the total effector repertoire. Dispensable effectors accounted for 15.5 ± 0.7%, and only a small fraction, 0.5 ± 0.1%, were classified as private (Supplementary Fig. 1a and b). These results indicate substantial intraspecific variability in effector content, while also suggesting the presence of a relatively stable core set across isolates.

### Comparative syntenic orthology reveals lineage-specific effector conservation in *P. theobromicola* and other cacao pathogens

To investigate patterns of gene conservation and diversification within *P. theobromicola*, we performed a syntenic orthology analysis using GENESPACE across 13 *Phytophthora* species, including the two long-read *P. theobromicola* primary assemblies (Supplementary Table 4). By incorporating the genomic context of orthologous groups, this approach enabled the detection of conserved gene blocks while accounting for structural rearrangements across genomes. Genes located within syntenic regions were classified into three categories: those conserved across all species, those conserved only among cacao-infecting species (cacao-infecting based on published reports), and those unique to *P. theobromicola*. A maximum-likelihood phylogenetic tree of the species in study (Supplementary Fig. 2) revealed that among the five cacao infecting pathogens, *P. theobromicola*, *P. capsici* and *P. citrophthora* were closely related while *P. megakarya* and *P palmivora* were more distant from the other three.

**Fig. 2.**
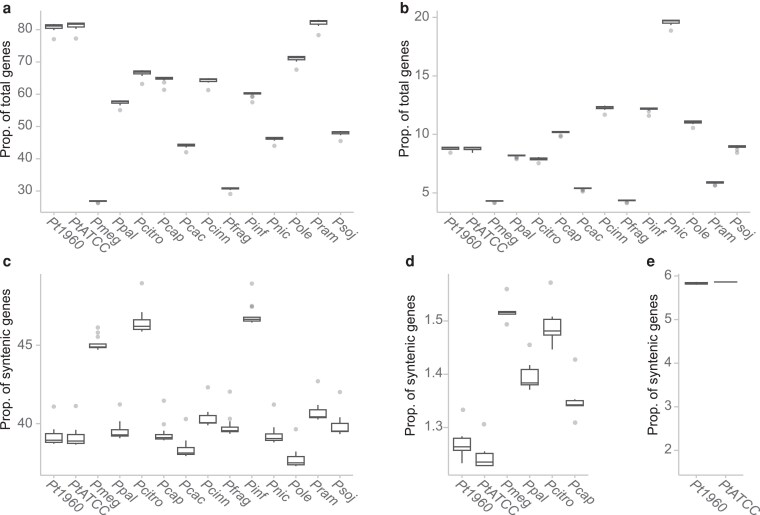
Syntenic orthologous gene groups in multiple *Phytophthora* species. a) Proportion of total genes with syntenic orthologs in at least one other species. b) Proportion of total genes derived from duplication of a syntenic ortholog. c) Proportion of syntenic orthologs conserved in all *Phytophthora* species in study. d) Proportion of syntenic orthologs conserved only in cacao pathogens. e) Proportion of syntenic orthologs conserved only in *P. theobromicola*. Pt1960: *P. t*h*eobromicola* MB01960, PtATCC: *P. t*h*eobromicola* P0449/ATCC, Pmeg: *P. m*e*gakarya*, Ppal: *P. p*a*lmivora*, Pcitro: *P.* c*itrophthora*, Pcap: *P.* c*apsici*, Pcac: *P.* c*actorum*, Pcinn: *P.* c*innamomi*, Pfrag: *P.* f*ragariae*, Pinf: *P.* i*nfestans*, Pnic: *P. n*i*cotianae*, Pole: *P. ol*e*ae*, Pram: *P. r*a*morum* and Psoj: *P. so*j*ae*.

Over 80% of genes in *P. theobromicola* have syntenic orthologs in at least one other *Phytophthora* species (Fig. 2a). Additionally, approximately 9% of the total gene content corresponds to local duplications of syntenic orthologs (Fig. 2b). These proportions contrast with species such as *P. megakarya* and *P. fragariae*, which have genome sizes and gene counts more than 2.5 times greater than those of *P. theobromicola*. From the total syntenic gene sets analyzed, an average of 40.8 ± 0.2% of genes are conserved across all species (Fig. 2c). In contrast, only 1.4 ± 0.02% are conserved exclusively among cacao-infecting *Phytophthora* species (Fig. 2d), while 5.8 ± 0.02% appear to be conserved only in *P. theobromicola* (Fig. 2e). These results highlight the presence of both highly conserved genes and a subset of lineage-specific genes that may contribute to host adaptation and species-specific functions.

**Fig. 3.**
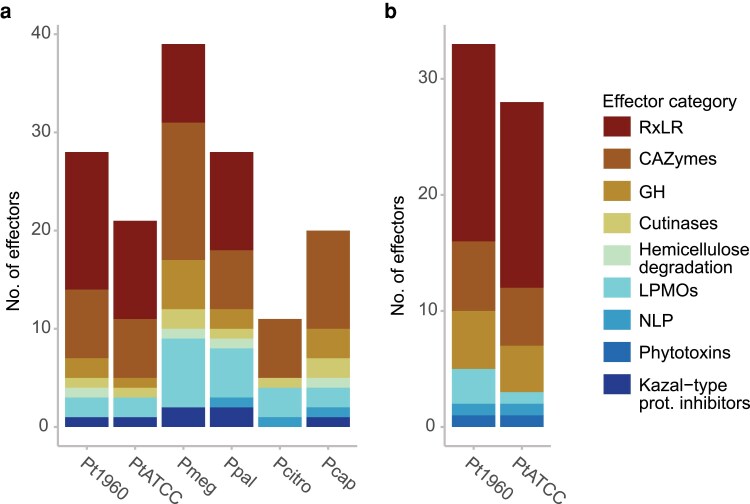
Number and function of secreted effectors classified as syntenic orthologs a) conserved only in cacao pathogens and b) conserved only in *P. theobromicola*. Panels a and b share the same color legend. Kazal-type prot. inhibitors only include the ones annotated as such in Table 2. Pt1960: *P. t*h*eobromicola* MB01960, PtATCC: *P. t*h*eobromicola* P0449/ATCC, Pmeg: *P. m*e*gakarya*, Ppal: *P. p*a*lmivora*, Pcitro: *P.* c*itrophthora*, Pcap: *P.* c*apsici*.

To further examine gene variability across *P. theobromicola* isolates, we intersected the syntenic ortholog categories with the graph-based classifications and secreted effector annotations. This integration enabled us to assess how conserved and lineage-specific genes contribute to the core and dispensable genomes, with a particular focus on effectors. On average, 5,165 ± 46 syntenic orthologs (SynOrs) conserved across all *Phytophthora* species were also classified as core genes in *P. theobromicola*, representing 34.8 ± 0.02% of the species' core genome based on the sequence graph (Supplementary Fig. 3a). In addition, 258 ± 44 universally conserved SynOrs were found in the dispensable category, comprising ∼21% of the dispensable gene set per genome. Among SynOrs conserved exclusively in cacao-infecting *Phytophthora* species, 175 ± 4 were core genes (1.2% of the total core genome), and 34 ± 5 were dispensable (2.75% of the dispensable set; Supplementary Fig. 3b). Similarly, SynOrs conserved only in *P. theobromicola* accounted for 4.8% of the total core genes and approximately 9.0% of the dispensable genes (Supplementary Fig. 3c). Effector genes followed similar patterns. On average, 10.5 ± 0.3% of effectors per genome were classified as SynOrs conserved across all species (Supplementary Fig. 4a), 2.3 ± 0.1% were conserved only among cacao pathogens (Supplementary Fig. 4b), and 4.3 ± 0.02% were conserved exclusively in *P. theobromicola* (Supplementary Fig. 4c). Within these groups, RxLR effectors and CAZymes were particularly well represented (Fig. 3, a and b), alongside other functional categories such as cutinases, hemicellulose-degrading enzymes, LPMOs, NLPs, and phytotoxins.

### Analysis of homology, duplication, and intergenic space highlights genomic drivers of effector variation

**Fig. 4.**
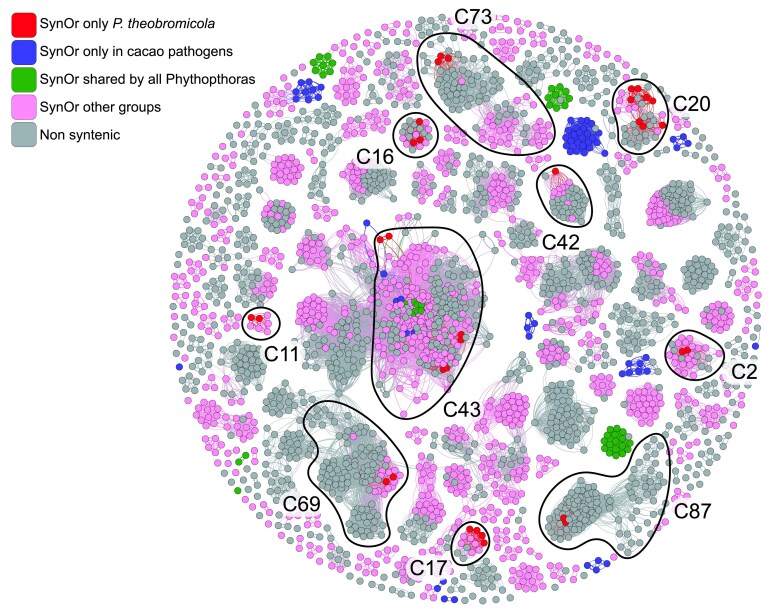
Homology network analysis of all the putative RxLR effectors among *phytophthora* species with focus on SynOr exclusive to *P. theobromicola*. Each RxLR is represented by a node. Edges connecting two nodes represent the similarity shared by the two proteins. Clusters were calculated based on the interconnectedness between proteins as described in the methods. Nodes are colored based on the syntenic orthology groups.

To investigate the evolutionary relationships and diversification patterns of RxLR effectors among cacao-infecting *Phytophthora* species, we performed a homology-based network analysis. All predicted RxLR protein sequences were compared using BLASTp, and the resulting similarity network was visualized and clustered using Gephi. This approach allowed the grouping of RxLRs into putative families based on shared sequence features, while also highlighting their SynOr categories as defined in the previous analyses. Several *P. theobromicola*-specific SynOrs clustered with RxLRs from multiple species, as well as with SynOrs conserved across broader taxonomic groups (Fig. 4). For each of the highlighted clusters in the network, protein sequences were aligned using MUSCLE (v5.3), and maximum likelihood phylogenetic trees were constructed (Supplementary File 1). Within each tree, SynOrs conserved exclusively in *P. theobromicola* consistently grouped together, forming distinct clades. The closest homologs to these SynOrs typically originated from *P. citrophthora* or *P. capsici*, and rarely from *P. megakarya* or *P. palmivora*. This pattern suggests that the observed variability may arise from lineage-specific duplications, gene rearrangements, or divergence from a shared ancestral gene set.

**Fig. 5.**
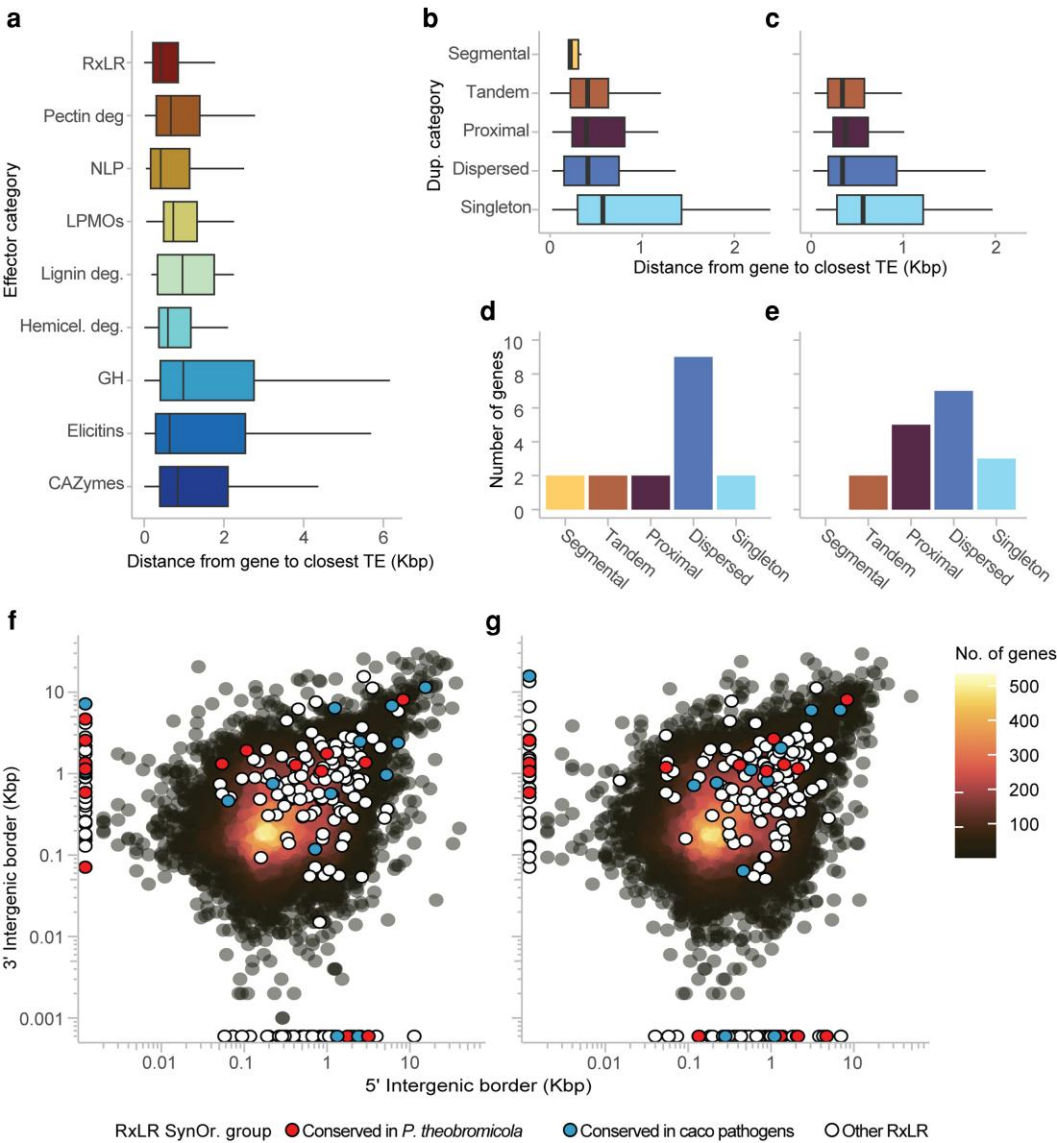
Classification of *P. theobromicola* effectors based on proximity to transposable elements (TEs) and intergenic space. a) Distance of different effector functional categories to the closest annotated TE. b–c) RxLR effectors classified by duplication category and their distance to the nearest TE in isolates MB01960 and P0449, respectively. d–e) Number of SynOr RxLRs conserved only in *P. theobromicola*, classified by duplication category in isolates MB01960 and P0449, respectively. f–g) Intergenic space surrounding genes in isolates MB01960 and P0449, respectively, highlighting RxLR effectors and colored by SynOr group classification. Panels f and g shares the same color legend.

Given the role of transposable elements (TEs) in promoting genome plasticity and effector diversification (Raffaele et al. 2010; Morales-Cruz et al. 2020), we examined the spatial distribution of RxLRs and other effector categories in relation to annotated Class I and Class II TEs in *P. theobromicola*. Genes located near TEs are frequently subject to duplication, insertional mutagenesis, and other forms of structural variation (Dong et al. 2015; Faino et al. 2016; Schrader and Schmitz 2019). In our analysis, RxLR effectors were found to be significantly closer to TEs than any other effector category, with the exception of NLPs (Kolmogorov–Smirnov test, *P* < 0.024; Fig. 5a).

To investigate the origins of RxLR variability, we conducted a duplication classification using MCScanX and assessed the proximity of each RxLR gene to nearby TEs. Across isolates, *P. theobromicola* harbored an average of 202 ± 6 RxLR effectors, of which 6 were derived from segmental duplications, 39 ± 2 from tandem duplications, 51 ± 2 from proximal duplications, 42 ± 1 from dispersed duplications, and 68 ± 2 were singletons. There were no statistically significant differences in the distribution of duplication categories between isolates (Kolmogorov–Smirnov test); however, segmental duplications were only detected in the MB01960 isolate (Fig. 5, b and c). Notably, most of the *P. theobromicola*-specific SynOr RxLRs were associated with dispersed and proximal duplications, whereas those classified as singletons frequently mapped near DNA transposable elements (Fig. 5, d and e). These findings support a role for both local duplication and TE-mediated mechanisms in shaping RxLR diversity in this species.

To further explore the sources of variability in the effector repertoire of *P. theobromicola*, we quantified intergenic distances surrounding all genes, with a focus on RxLR effectors. Larger intergenic regions have been associated with relaxed selective constraints and increased potential for sequence diversification through mutation or structural rearrangement (Raffaele and Kamoun 2012; Dong et al. 2015). Genes with secretion signal exhibited significantly larger intergenic distances compared to all other genes (Wilcoxon rank-sum test, *P* < 2.2 x 10⁻¹⁶), with a mean intergenic distance of 916 ± 24 bp, in contrast to 684 ± 9 bp for non-secreted genes (Supplementary Fig. 5a and b). Similarly, RxLR genes exhibited significantly larger intergenic distances compared to all other genes (Wilcoxon rank-sum test, *P* < 2.2 x 10⁻¹⁶), with a mean intergenic distance of 1,401 ± 74 bp, in contrast to 697 ± 6 bp for non-RxLR genes (Fig. 5, f and g). Furthermore, SynOr RxLRs conserved exclusively in cacao-infecting species and those unique to *P. theobromicola* displayed similar intergenic lengths, both significantly greater than those of the remaining RxLRs (Dunn's test, *P* < 0.01; Fig. 5, f and g). These results suggest that the broader intergenic context of these effectors may facilitate their diversification, particularly for lineage-specific RxLRs, reinforcing the role of genomic architecture in shaping effector evolution.

### Expression profiling reveals in-planta activation of effectors including the lineage-specific genes

While gene prediction provides a useful overview of the genomic potential of a species, expression profiling helps determine which genes are potentially active under biologically relevant conditions. To assess the *in planta* expression of *P. theobromicola* genes during infection, we inoculated three different cacao tissues, pods, leaves, and stems, with mycelial plugs from the P0449 isolate and performed RNA-seq analysis alongside matched uninoculated controls. Among the infected tissues, pods exhibited the highest number of expressed *P. theobromicola* genes, followed by leaves and stems (Supplementary Fig. 6a–c). About 88% of the predicted effector repertoire was expressed in at least one tissue. Pods showed the highest expression across all effector categories, with approximately 70% of predicted RxLRs, 100% of Secreted CRN (∼76% of non-secretion signal CRN), and 90% of effector-associated CAZymes detected in the transcriptome (Supplementary Fig. 7).

**Fig. 6.**
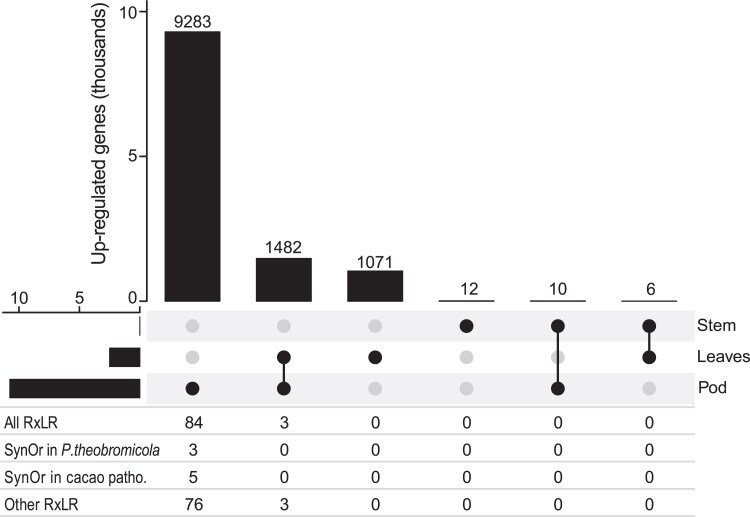
Comparison of up-regulated genes per inoculated tissue. The table presents the number of RxLR effectors in each bar including the total set of RxLRs and the RxLRs in the different SynOr categories.

Differential expression analysis revealed tissue-specific responses. A total of 9,283 *P. theobromicola* genes were significantly upregulated in pods relative to both leaves and stems (log₂FC > 1; adjusted *P*-value < 0.05). Additionally, 1,482 genes were upregulated in both pods and leaves compared to stems, and 1,071 genes were specifically upregulated in leaves. In contrast, very few genes were preferentially expressed in stems (Fig. 6). Among the genes upregulated exclusively in pods, 84 were RxLR effectors, including three classified as SynOrs conserved only in *P. theobromicola* and five conserved only among cacao-infecting species (Fig. 6). These results suggest tissue-specific effector deployment, with pods being a major site of effector activity during infection.

**Fig. 7.**
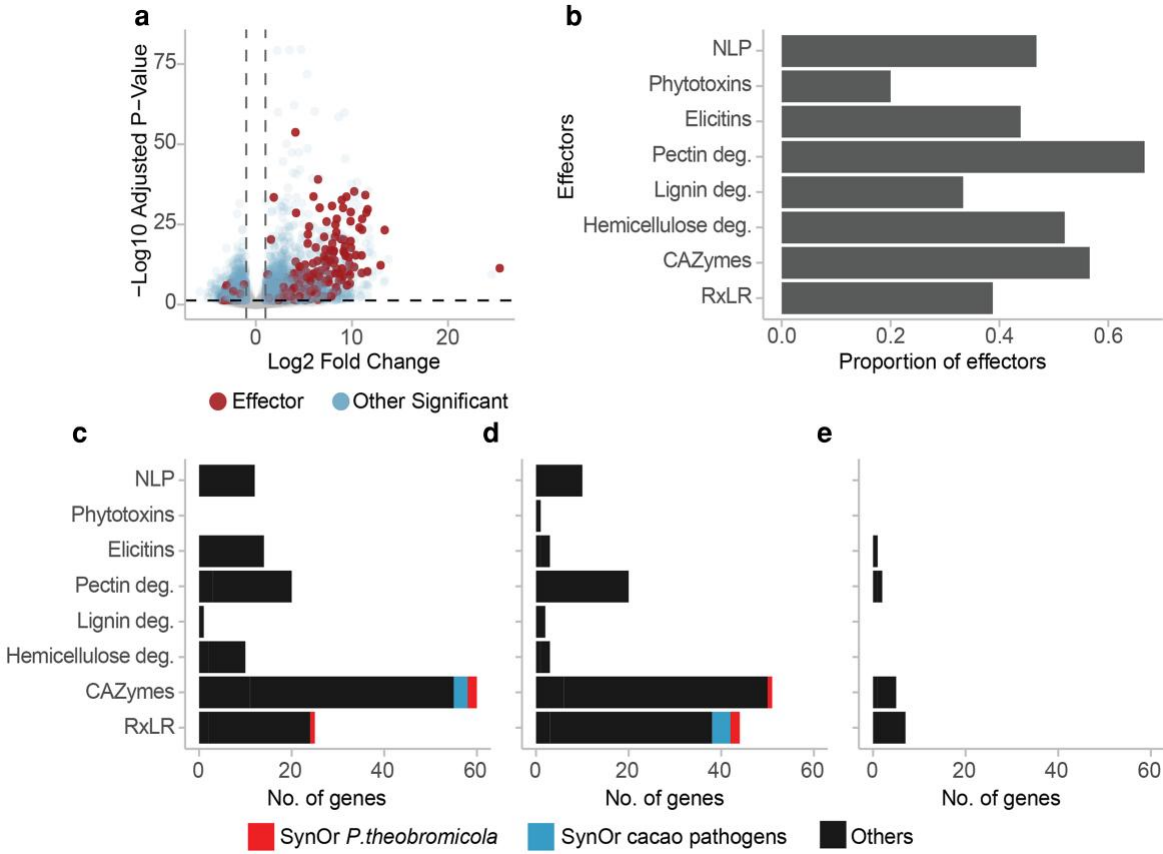
Differential expression of P*. theobromicola* genes *in planta* compared to *in vitro*. a) Volcano plot showing differentially expressed genes between *in planta* and *in vitro* conditions, highlighting effectors. b) Proportion of total effectors from each functional category upregulated *in planta*. c–e) Number of genes with Log2 fold change (L2FC) values between 1–5 (c), 5–10 (d), and 10–15 (e). Panels c–e share the same color legend.

Gene expression in inoculated tissues was compared to pure mycelial cultures to identify differentially expressed (DE) genes. A total of 5,108 genes were differentially expressed, with 63% upregulated *in planta* (Fig. 7a), including 46% of the predicted effector repertoire (Fig. 7b). Among these, RxLR effectors and CAZymes showed a high proportion of genes with log₂ fold changes (L2FC) between 1 and 5 (Fig. 7c). Interestingly, the number of RxLR genes with L2FC values between 5 and 10 was nearly double that of the previous range and included two SynOrs conserved exclusively in *P. theobromicola* (Fig. 7d). Two out of the three *P. theobromicola*-specific SynOr RxLRs classified as singletons were strongly upregulated *in planta*, showing 5-fold and 53-fold higher expression, respectively, compared to *in vitro* mycelial samples. A smaller subset of effector genes exhibited even more extreme differential expression, with L2FC values between 10 and 15 (Fig. 7e). These findings suggest strong host-induced expression of key effectors, including lineage-specific genes that may play critical roles in pathogenicity and host adaptation.

## Discussion

*Phytophthora theobromicola* has recently emerged as a major contributor to black pod rot disease in Brazil, exhibiting levels of aggressiveness comparable to those of *P. palmivora* (Decloquement et al. 2021). Despite the natural difficulty of assembling *Phytophthora* genomes due to their high repeat content (Cox et al. 2022), here, we report high-quality and high-completeness genome assemblies generated from multiple *P. theobromicola* isolates. The assembled genome size and number of predicted protein-coding genes are similar to those of its close relative *P. citrophthora*. This finding is consistent with earlier reports of misidentification of *P. theobromicola* as *P. citrophthora* because of their morphological similarities (Luz et al. 2018; Decloquement et al. 2021).

Access to high-quality genome assemblies enabled the annotation of putative effector genes in *P. theobromicola*. Approximately 45% of the predicted secretome in the assembled genomes was assigned to known effector categories. While this proportion is comparable to that observed in other *Phytophthora* species infecting cacao, the total number of effectors in *P. theobromicola* is considerably lower than in species such as *P. megakarya* and *P. palmivora*. This discrepancy is partially explained by the whole-genome duplications previously reported in *P. megakarya* and *P. palmivora*, which resulted in expanded gene content and two of the largest effector repertoires within the genus (Morales-Cruz et al. 2020).

Nonetheless, the repertoire of virulence factors, and particularly effectors, can vary substantially among isolates, as demonstrated in previous studies of filamentous pathogens (Morales-Cruz et al. 2020; Garcia et al. 2024). To better capture this intraspecific variation, we constructed a sequence graph that integrates the genomes of multiple *P. theobromicola* isolates, enabling a more comprehensive view of the species' effector complement. The marked increase in the proportion of dispensable genes and effectors, along with the finding that approximately 20% of predicted effectors are dispensable, indicates a high degree of genomic variability in *P. theobromicola*. Similar patterns have been reported in other highly virulent filamentous pathogens (Garcia et al. 2024). The relatively slow growth of the total effector repertoire with the addition of new genomes (Fig. 1c) suggests that while novel genomes continue to contribute new genes, their greatest impact is on the dispensable and core proportions, a pattern that could be driven by the intrinsic genomic plasticity of *Phytophthora* species (Kronmiller et al. 2023). It is important to note that the sequence graph approach may be influenced by the contiguity and completeness of the input assemblies. This effect is most pronounced in repetitive regions but can also impact a small fraction of genes interrupted by contig breaks in short-read assemblies. Additionally, characterizing virulence differences among these isolates would provide valuable context for interpreting the observed genomic variation. Such phenotypic comparisons were not performed in this study but should be addressed in future work.

Syntenic orthology analysis provided a framework to further explore genomic flexibility and track patterns of effector diversification in *P. theobromicola*. By comparing a diverse set of *Phytophthora* species with varying host ranges, we identified syntenic ortholog groups conserved across all species, conserved only among cacao-infecting pathogens, and unique to *P. theobromicola*. Within the effector genes falling into cacao-specific and *P. theobromicola*-specific SynOr categories, RxLRs and CAZymes were particularly enriched. RxLR effectors, in particular, have been consistently associated with expanded intergenic regions and elevated evolutionary rates compared to other genomic regions in *Phytophthora* (Raffaele et al. 2010; Stassen and Van den Ackerveken 2011; Dong et al. 2015; Morales-Cruz et al. 2020). These characteristics likely contribute to the high variability in orthology and disrupted collinearity observed even among species within the same genus. It is also important to note that the apparent conservation of effectors in cacao-infecting pathogens may reflect a combination of host-driven selective pressures and shared phylogenetic history. Additional experiments will be needed to disentangle and confirm the relative contribution of each factor.

The relationship between RxLR effectors and SynOr gene classification was further explored through a homology-based network including RxLR sequences from multiple cacao-infecting *Phytophthora* species. Clustering patterns and phylogenetic relationships within these groups suggest that SynOrs conserved exclusively in *P. theobromicola* likely originated through lineage-specific duplication or translocation events. Previous studies have shown that such genomic variability is often associated with the activity of transposable elements (TEs), which can drive effector diversification and structural genome variation (Raffaele and Kamoun 2012; Faino et al. 2016; Schrader and Schmitz 2019; Morales-Cruz et al. 2020; Zhang et al. 2024). Consistent with this, RxLRs and a subset of other effectors in *P. theobromicola* are located significantly closer to TEs than CAZymes or non-effector genes, mirroring patterns previously reported in other *Phytophthora* pathogens of cacao (Morales-Cruz et al. 2020).

Moreover, SynOr RxLRs conserved only in *P. theobromicola* that do not arise from duplication events (i.e. singletons) are frequently located near DNA transposable elements. Given that DNA elements typically follow a cut-and-paste mechanism (Wicker et al. 2007), these RxLRs were likely translocated from other genomic regions (Raffaele and Kamoun 2012; Schrader and Schmitz 2019), resulting in structural rearrangements unique to *P. theobromicola*. The larger intergenic distances observed around RxLR genes, compared to other genes, are consistent with the two-speed genome model, in which effector genes, particularly RxLRs, reside in gene-sparse, repeat-rich regions that evolve more rapidly and contribute to host adaptation (Dong et al. 2015; Frantzeskakis et al. 2019). Notably, many RxLRs were located near scaffold ends, a pattern likely caused by large intergenic, repeat-rich regions associated with RxLRs that complicate assembly and frequently result in contig breaks. In fungal pathogens, subtelomeric regions are known hotspots for effector variability due to high recombination rates (Croll et al. 2015; Diotti et al. 2021; Sauters and Rokas 2025). However, we could not confidently determine whether this is also the case in the oomycete *P. theobromicola*, as previously reported telomeric repeats were not detected at both ends of our scaffolds. This limitation may stem from the sequencing technologies used in this study.

Gene expression analysis of *P. theobromicola* in different inoculated cacao tissues revealed that at least 88% of the predicted effector genes are transcriptionally active, including 70% of the total predicted RxLRs expressed in pods. This proportion of active RxLRs is comparable to that observed in *P. megakarya* and *P. palmivora*, where approximately 75% of RxLRs are expressed *in planta* (Morales-Cruz et al. 2020). Pods appeared to be the major site of pathogen activity, as most upregulated genes were specific to this tissue. Among them were 87 RxLRs, including six classified as SynOrs conserved exclusively in *P. theobromicola* or among cacao-infecting *Phytophthora* species. These findings highlight pods as a key infection site and suggest a specialized deployment of effectors, particularly lineage-specific RxLRs, during host colonization. Nonetheless, recognize that the validation of these effectors would have been a valuable addition to the paper. However, it is outside the scope of this study, should be considered for future projects studying these candidate effectors.

Comparison of *P. theobromicola* gene expression *in planta* and *in vitro* revealed that the majority of differentially expressed genes were upregulated during host colonization, including most predicted effectors. Only a small subset of effectors was upregulated *in vitro*, suggesting that effector expression is primarily induced by host-derived cues and likely regulated in response to plant signals (Giraldo and Valent 2013; Inoue et al. 2023). This expression pattern is consistent with the established role of effectors in modulating plant immune responses, reinforcing their functional importance during infection. Importantly, several SynOr RxLRs conserved exclusively in *P. theobromicola* were also transcriptionally active *in planta*, indicating that these lineage-specific genes are not only structurally unique but also likely contribute to pathogenicity.

This study presents the first comprehensive genomic analysis of the black pod rot pathogen *Phytophthora theobromicola*. Our results reveal a highly flexible genome, shaped by structural rearrangements and enriched for variable effector genes, many of which are closely associated with transposable elements. Integration of sequence graph analyses across multiple isolates, synteny comparisons, and transcriptomic profiling demonstrates that numerous lineage-specific RxLR effectors are actively expressed in cacao pods and are frequently located in gene-sparse, repeat-rich regions—consistent with the two-speed genome model. Together, these findings highlight the role of genome plasticity in effector diversification and potentially host adaptation, and provide a foundation for future investigations into the evolutionary dynamics and pathogenic strategies of *P. theobromicola*.

## Data Availability

The sequencing data and genome assemblies for this project are available at NCBI (https://www.ncbi.nlm.nih.gov/bioproject/PRJNA1257449) The genome assemblies and gene models produced in this study are also publicly available at Zenodo (10.5281/zenodo.15346916). Dedicated genome browser and BLAST tools are available at cacaopathogenomics.com. Supplementary material available at G3 FigShare: https://doi.org/10.25387/g3.30291553.

## References

[jkaf256-B1] Appiah AA, Flood J, Archer SA, Bridge PD. 2004. Molecular analysis of the major Phytophthora species on cocoa. [updated 2025 April 14]. https://bsppjournals.onlinelibrary.wiley.com/doi/10.1111/j.0032-0862.2004.00980.x.

[jkaf256-B2] Argout X et al 2017. The cacao Criollo genome v2.0: an improved version of the genome for genetic and functional genomic studies. BMC Genomics. 18:730. 10.1186/s12864-017-4120-9. 28915793 PMC5603072

[jkaf256-B3] Barnett DW et al 2011. BamTools: a C++ API and toolkit for analyzing and managing BAM files. Bioinformatics. 27:1691–1692. 10.1093/bioinformatics/btr174. 21493652 PMC3106182

[jkaf256-B4] Bastian M, Heymann S, Jacomy M. 2009. Gephi: an open source software for exploring and manipulating networks. Proc Int AAAI Conf Weblogs Soc Media. 3:361–362. 10.1609/icwsm.v3i1.13937.

[jkaf256-B5] Boetzer M, Pirovano W. 2014. SSPACE-LongRead: scaffolding bacterial draft genomes using long read sequence information. BMC Bioinformatics. 15:211. 10.1186/1471-2105-15-211. 24950923 PMC4076250

[jkaf256-B6] Bolger AM, Lohse M, Usadel B. 2014. Trimmomatic: a flexible trimmer for Illumina sequence data. Bioinformatics. 30:2114–2120. 10.1093/bioinformatics/btu170. 24695404 PMC4103590

[jkaf256-B7] Brůna T et al 2021. BRAKER2: automatic eukaryotic genome annotation with GeneMark-EP+ and AUGUSTUS supported by a protein database. NAR Genom Bioinform. 3:lqaa108. 10.1093/nargab/lqaa108. 33575650 PMC7787252

[jkaf256-B8] Brůna T, Lomsadze A, Borodovsky M. 2020. GeneMark-EP+: eukaryotic gene prediction with self-training in the space of genes and proteins. NAR Genom Bioinform. 2:lqaa026. 10.1093/nargab/lqaa026. 32440658 PMC7222226

[jkaf256-B9] Buchfink B, Reuter K, Drost H-G. 2021. Sensitive protein alignments at tree-of-life scale using DIAMOND. Nat Methods. 18:366–368. 10.1038/s41592-021-01101-x. 33828273 PMC8026399

[jkaf256-B10] Buchfink B, Xie C, Huson DH. 2015. Fast and sensitive protein alignment using DIAMOND. Nat Methods. 12:59–60. 10.1038/nmeth.3176. 25402007

[jkaf256-B11] Chao K-H et al 2024. Combining DNA and protein alignments to improve genome annotation with LiftOn. Genome Res. 35:311–325. 10.1101/gr.279620.124. PMC11874971 39730188

[jkaf256-B12] Chin C-S et al 2016. Phased diploid genome assembly with single-molecule real-time sequencing. Nat Methods. 13:1050–1054. 10.1038/nmeth.4035. 27749838 PMC5503144

[jkaf256-B13] Cox MP et al 2022. Chromosome-level assembly of the *Phytophthora agathidicida* genome reveals adaptation in effector gene families. Front Microbiol. 13:1038444. 10.3389/fmicb.2022.1038444. 36406440 PMC9667082

[jkaf256-B14] Croll D, Lendenmann MH, Stewart E, McDonald BA. 2015. The impact of recombination hotspots on genome evolution of a fungal plant pathogen. Genetics. 201:1213–1228. 10.1534/genetics.115.180968. 26392286 PMC4649646

[jkaf256-B15] Decloquement J et al 2021. *Phytophthora theobromicola* sp. nov.: a new species causing black pod disease on Cacao in Brazil. Front Microbiol. 12:537399. 10.3389/fmicb.2021.537399. 33815301 PMC8015942

[jkaf256-B16] de Oliveira ML . 2005. Identificação e manejo das principais doenças do cacaueiro no Brasil. Seção de fitopatologia; Centro de pesquias de cacau; Comissão executiva do plano da lavoura cacaueira.

[jkaf256-B17] Diotti R, Esposito M, Shen CH. 2021. Telomeric and sub-telomeric structure and implications in fungal opportunistic pathogens. Microorganisms. 9:1405. 10.3390/microorganisms9071405. 34209786 PMC8305976

[jkaf256-B18] Dong S, Raffaele S, Kamoun S. 2015. The two-speed genomes of filamentous pathogens: waltz with plants. Curr Opin Genet Dev. 35:57–65. 10.1016/j.gde.2015.09.001. 26451981

[jkaf256-B19] Dou D et al 2008. RXLR-Mediated Entry of *Phytophthora sojae* effector *Avr1b* into soybean cells does not require pathogen-encoded machinery. Plant Cell. 20:1930–1947. 10.1105/tpc.107.056093. 18621946 PMC2518231

[jkaf256-B20] Drenth A, Guest D. 2013. Phytophthora palmivora in tropical tree crops. In: Lamour K, editors. Phytophthora: a global perspective. 1st ed. CABI. p. 187–196.

[jkaf256-B21] Dyrløv Bendtsen J, Nielsen H, von Heijne G, Brunak S. 2004. Improved prediction of signal peptides: SignalP 3.0. J Mol Biol. 340:783–795. 10.1016/j.jmb.2004.05.028. 15223320

[jkaf256-B22] Edgar RC . 2022. Muscle5: high-accuracy alignment ensembles enable unbiased assessments of sequence homology and phylogeny. Nat Commun. 13:6968. 10.1038/s41467-022-34630-w. 36379955 PMC9664440

[jkaf256-B23] Emms DM, Kelly S. 2019. OrthoFinder: phylogenetic orthology inference for comparative genomics. Genome Biol. 20:238. 10.1186/s13059-019-1832-y. 31727128 PMC6857279

[jkaf256-B24] Faino L et al 2016. Transposons passively and actively contribute to evolution of the two-speed genome of a fungal pathogen. Genome Res. 26:1091–1100. 10.1101/gr.204974.116. 27325116 PMC4971763

[jkaf256-B25] Firman ID, Vernon AJ. 1970. Cocoa canker caused by *Phytophthora palmivora*. Ann Appl Biol. 65:65–73. 10.1111/j.1744-7348.1970.tb04563.x.

[jkaf256-B26] Flynn JM et al 2020. RepeatModeler2 for automated genomic discovery of transposable element families. Proc Natl Acad Sci USA. 117:9451–9457. 10.1073/pnas.1921046117. 32300014 PMC7196820

[jkaf256-B27] Frantzeskakis L, Kusch S, Panstruga R. 2019. The need for speed: compartmentalized genome evolution in filamentous phytopathogens. Mol Plant Pathol. 20:3–7. 10.1111/mpp.12738. 30557450 PMC6430476

[jkaf256-B28] Fruchterman TMJ, Reingold EM. 1991. Graph drawing by force-directed placement. Softw Pract Exp. 21:1129–1164. 10.1002/spe.4380211102.

[jkaf256-B29] Garcia JF et al 2024. Comparative pangenomic insights into the distinct evolution of virulence factors among grapevine trunk pathogens. Mol Plant Microbe Interact. 37:127–142. 10.1094/MPMI-09-23-0129-R. 37934016

[jkaf256-B30] Giraldo MC, Valent B. 2013. Filamentous plant pathogen effectors in action. Nat Rev Microbiol. 11:800–814. 10.1038/nrmicro3119. 24129511

[jkaf256-B31] Gotoh O . 2008. A space-efficient and accurate method for mapping and aligning cDNA sequences onto genomic sequence. Nucleic Acids Res. 36:2630–2638. 10.1093/nar/gkn105. 18344523 PMC2377433

[jkaf256-B32] Guest D . 2007. Black pod: diverse pathogens with a global impact on cocoa yield. Phytopathology. 97:1650–1653. 10.1094/PHYTO-97-12-1650. 18943728

[jkaf256-B33] Heumos S et al 2024. Cluster-efficient pangenome graph construction with nf-core/pangenome. Bioinformatics. 40:btae609. 10.1093/bioinformatics/btae609. 39400346 PMC11568064

[jkaf256-B34] Hoff KJ et al 2016. BRAKER1: unsupervised RNA-Seq-based genome annotation with GeneMark-ET and AUGUSTUS. Bioinformatics. 32:767–769. 10.1093/bioinformatics/btv661. 26559507 PMC6078167

[jkaf256-B35] Hoff KJ, Lomsadze A, Borodovsky M, Stanke M. 2019. Whole-genome annotation with BRAKER. In: Kollmar M, editors. Gene prediction: methods and protocols. Springer. p. 65–95. 10.1007/978-1-4939-9173-0_5 PMC6635606 31020555

[jkaf256-B36] Inoue Y et al 2023. Selective deployment of virulence effectors correlates with host specificity in a fungal plant pathogen. New Phytol. 238:1578–1592. 10.1111/nph.18790. 36939621

[jkaf256-B37] Iwata H, Gotoh O. 2012. Benchmarking spliced alignment programs including Spaln2, an extended version of Spaln that incorporates additional species-specific features. Nucleic Acids Res. 40:e161. 10.1093/nar/gks708. 22848105 PMC3488211

[jkaf256-B38] Jiang RHY, Tyler BM. 2012. Mechanisms and evolution of virulence in oomycetes. Annu Rev Phytopathol. 50:295–318. 10.1146/annurev-phyto-081211-172912. 22920560

[jkaf256-B39] Kamoun S . 2006. A catalogue of the effector secretome of plant pathogenic oomycetes. Annu Rev Phytopathol. 44:41–60. 10.1146/annurev.phyto.44.070505.143436. 16448329

[jkaf256-B40] Kim D et al 2019. Graph-based genome alignment and genotyping with HISAT2 and HISAT-genotype. Nat Biotechnol. 37:907–915. 10.1038/s41587-019-0201-4. 31375807 PMC7605509

[jkaf256-B41] Kozlov AM et al 2019. RAxML-NG: a fast, scalable and user-friendly tool for maximum likelihood phylogenetic inference. Bioinformatics. 35:4453–4455. 10.1093/bioinformatics/btz305. 31070718 PMC6821337

[jkaf256-B42] Krogh A, Larsson B, von Heijne G, Sonnhammer ELL. 2001. Predicting transmembrane protein topology with a hidden Markov model: application to complete genomes1. J Mol Biol. 305:567–580. 10.1006/jmbi.2000.4315. 11152613

[jkaf256-B43] Kronmiller BA et al 2023. Comparative genomic analysis of 31 *Phytophthora* genomes reveals genome plasticity and horizontal gene transfer. Mol Plant Microbe Interact. 36:26–46. 10.1094/MPMI-06-22-0133-R. 36306437

[jkaf256-B44] Kuznetsov D et al 2023. OrthoDB v11: annotation of orthologs in the widest sampling of organismal diversity. Nucleic Acids Res. 51:D445–D451. 10.1093/nar/gkac998. 36350662 PMC9825584

[jkaf256-B45] Lenarčič T et al 2017. Eudicot plant-specific sphingolipids determine host selectivity of microbial NLP cytolysins. Science. 358:1431–1434. 10.1126/science.aan6874. 29242345

[jkaf256-B46] Li H et al 2009. The sequence alignment/map format and SAMtools. Bioinformatics. 25:2078–2079. 10.1093/bioinformatics/btp352. 19505943 PMC2723002

[jkaf256-B47] Liu T et al 2011. Two host cytoplasmic effectors are required for pathogenesis of *Phytophthora sojae* by suppression of host defenses. Plant Physiol. 155:490–501. 10.1104/pp.110.166470. 21071601 PMC3075790

[jkaf256-B48] Lomsadze A, Burns PD, Borodovsky M. 2014. Integration of mapped RNA-Seq reads into automatic training of eukaryotic gene finding algorithm. Nucleic Acids Res. 42:e119. 10.1093/nar/gku557. 24990371 PMC4150757

[jkaf256-B49] Lomsadze A, Ter-Hovhannisyan V, Chernoff YO, Borodovsky M. 2005. Gene identification in novel eukaryotic genomes by self-training algorithm. Nucleic Acids Res. 33:6494–6506. 10.1093/nar/gki937. 16314312 PMC1298918

[jkaf256-B50] Love MI, Huber W, Anders S. 2014. Moderated estimation of fold change and dispersion for RNA-Seq data with DESeq2. Genome Biol. 15:550. 10.1186/s13059-014-0550-8. 25516281 PMC4302049

[jkaf256-B51] Lovell JT et al 2022. GENESPACE tracks regions of interest and gene copy number variation across multiple genomes. eLife. 11:e78526. 10.7554/eLife.78526. 36083267 PMC9462846

[jkaf256-B52] Luz EDMNM et al 2018. Influência do sistema de cultivo na biogeografia das espécies de Phytophthora patogênicas ao cacaueiro na Bahia, Brasil. Ministry of Agriculture, Livestock and Supply. p. 205–214.

[jkaf256-B53] Ma Z et al 2015. A *Phytophthora sojae* glycoside hydrolase 12 protein is a Major virulence factor during soybean infection and is recognized as a PAMP. Plant Cell. 27:2057–2072. 10.1105/tpc.15.00390. 26163574 PMC4531360

[jkaf256-B54] Manni M et al 2021. BUSCO update: novel and streamlined workflows along with broader and deeper phylogenetic coverage for scoring of eukaryotic, prokaryotic, and viral genomes. Mol Biol Evol. 38:4647–4654. 10.1093/molbev/msab199. 34320186 PMC8476166

[jkaf256-B55] Marelli J-P et al 2019. Chocolate under threat from old and new Cacao diseases. Phytopathology. 109:1331–1343. 10.1094/PHYTO-12-18-0477-RVW. 31115251

[jkaf256-B56] McGowan J, Fitzpatrick DA. 2017. Genomic, network, and phylogenetic analysis of the oomycete effector arsenal. mSphere. 2:e00408–e00417. 10.1128/msphere.00408-17. 29202039 PMC5700374

[jkaf256-B57] Minio A et al 2019. Diploid genome assembly of the wine grape carménère. G3 (Bethesda). 9:1331–1337. 10.1534/g3.119.400030. 30923135 PMC6505170

[jkaf256-B58] Möller H, Coetzee B, van Niekerk J, Rose LJ. 2024. Reference genome resource for the Citrus pathogen *Phytophthora citrophthora*. J Genomics. 12:14–18. 10.7150/jgen.89324. 38164508 PMC10751748

[jkaf256-B59] Morales-Cruz A et al 2020. Independent whole-genome duplications define the architecture of the genomes of the devastating west African Cacao black pod pathogen *Phytophthora megakarya* and its close relative *Phytophthora palmivora*. G3 (Bethesda). 10:2241–2255. 10.1534/g3.120.401014. 32354704 PMC7341134

[jkaf256-B60] Nur M, Wood K, Michelmore R. 2023. Effectoro: motif-independent prediction of effectors in oomycete genomes using machine learning and lineage specificity. Mol Plant Microbe Interact. 36:397–410. 10.1094/MPMI-11-22-0236-TA. 36853198

[jkaf256-B61] Paradis E, Schliep K. 2019. Ape 5.0: an environment for modern phylogenetics and evolutionary analyses in R. Bioinformatics. 35:526–528. 10.1093/bioinformatics/bty633. 30016406

[jkaf256-B62] Prjibelski A et al 2020. Using SPAdes De Novo assembler. Curr Protoc Bioinformatics. 70:e102. 10.1002/cpbi.102. 32559359

[jkaf256-B63] Puig AS et al 2018. The differential influence of temperature on *Phytophthora megakarya* and *Phytophthora palmivora* pod lesion expansion, mycelia growth, gene expression, and metabolite profiles. Physiol Mol Plant Pathol. 102:95–112. 10.1016/j.pmpp.2017.12.002.

[jkaf256-B64] Puig AS et al 2021. Phytophthora palmivora causing disease on Theobroma cacao in Hawaii. Agriculture. 11:396. 10.3390/agriculture11050396.

[jkaf256-B65] Quinlan AR . 2014. BEDTools: the Swiss-army tool for genome feature analysis. Curr Protoc Bioinformatics. 47:11.12.1–11.12.34. 10.1002/0471250953.bi1112s47. PMC4213956 25199790

[jkaf256-B66] Raffaele S et al 2010. Genome evolution following host jumps in the Irish potato famine pathogen lineage. Science. 330:1540–1543. 10.1126/science.1193070. 21148391

[jkaf256-B67] Raffaele S, Kamoun S. 2012. Genome evolution in filamentous plant pathogens: why bigger can be better. Nat Rev Microbiol. 10:417–430. 10.1038/nrmicro2790. 22565130

[jkaf256-B68] Revell LJ . 2024. Phytools 2.0: an updated R ecosystem for phylogenetic comparative methods (and other things). PeerJ. 12:e16505. 10.7717/peerj.16505. 38192598 PMC10773453

[jkaf256-B69] Rhie A, Walenz BP, Koren S, Phillippy AM. 2020. Merqury: reference-free quality, completeness, and phasing assessment for genome assemblies. Genome Biol. 21:245. 10.1186/s13059-020-02134-9. 32928274 PMC7488777

[jkaf256-B70] Sauters TJC, Rokas A. 2025. Patterns and mechanisms of fungal genome plasticity. Curr Biol. 35:R527–R544. 10.1016/j.cub.2025.04.003. 40494309 PMC12161452

[jkaf256-B71] Schmidt JE, Puig AS, DuVal AE, Pfeufer EE. 2023. Phyllosphere microbial diversity and specific taxa mediate within-cultivar resistance to *Phytophthora palmivora* in cacao. mSphere. 8:e00013–e00023. 10.1128/msphere.00013-23. 37603690 PMC10597403

[jkaf256-B72] Schornack S et al 2010. Ancient class of translocated oomycete effectors targets the host nucleus. Proc Natl Acad Sci U S A. 107:17421–17426. 10.1073/pnas.1008491107. 20847293 PMC2951462

[jkaf256-B73] Schrader L, Schmitz J. 2019. The impact of transposable elements in adaptive evolution. Mol Ecol. 28:1537–1549. 10.1111/mec.14794. 30003608

[jkaf256-B74] Shumate A, Salzberg SL. 2021. Liftoff: accurate mapping of gene annotations. Bioinformatics. 37:1639–1643. 10.1093/bioinformatics/btaa1016. 33320174 PMC8289374

[jkaf256-B75] Smit AFA, Hubley R, Green P. 2015. Repeatmodeler open-1.0. 2008–2015. Institute for Systems Biology.

[jkaf256-B76] Smit AFA, Hubley R, Green P. 2025. Repeatmasker [updated 2025 Apr 28]. https://www.repeatmasker.org.

[jkaf256-B77] Sperschneider J et al 2015. Evaluation of secretion prediction highlights differing approaches needed for oomycete and fungal effectors. Front Plant Sci. 6:1168. 10.3389/fpls.2015.01168. 26779196 PMC4688413

[jkaf256-B78] Stajich JE et al 2021. High-quality reference genome sequence for the oomycete vegetable pathogen phytophthora capsici strain LT1534. Microbiol Resour Announc. 10:e0029521. 10.1128/mra.00295-21. 34042486 PMC8201633

[jkaf256-B79] Stam R et al 2013. Identification and characterisation CRN effectors in phytophthora capsici shows modularity and functional diversity. PLoS One. 8:e59517. 10.1371/journal.pone.0059517. 23536880 PMC3607596

[jkaf256-B80] Stanke M, Diekhans M, Baertsch R, Haussler D. 2008. Using native and syntenically mapped cDNA alignments to improve *de novo* gene finding. Bioinformatics. 24:637–644. 10.1093/bioinformatics/btn013. 18218656

[jkaf256-B81] Stanke M, Schöffmann O, Morgenstern B, Waack S. 2006. Gene prediction in eukaryotes with a generalized hidden Markov model that uses hints from external sources. BMC Bioinformatics. 7:62. 10.1186/1471-2105-7-62. 16469098 PMC1409804

[jkaf256-B82] Stassen JH, Van den Ackerveken G. 2011. How do oomycete effectors interfere with plant life? Curr Opin Plant Biol. 14:407–414. 10.1016/j.pbi.2011.05.002. 21641854

[jkaf256-B83] Surujdeo-Maharaj S, Sreenivasan TN, Motilal LA, Umaharan P. 2016. Black pod and other phytophthora induced diseases of Cacao: history, biology, and control. In: Bailey BA, Meinhardt LW, editors. Cacao diseases: a history of old enemies and new encounters. Springer. p. 213–266.

[jkaf256-B84] Tabima JF, Grünwald NJ. 2019. *Effectr*: an expandable R package to predict candidate RxLR and CRN effectors in oomycetes using motif searches. Mol Plant Microbe Interact. 32:1067–1076. 10.1094/MPMI-10-18-0279-TA. 30951442

[jkaf256-B85] Ter-Hovhannisyan V, Lomsadze A, Chernoff YO, Borodovsky M. 2008. Gene prediction in novel fungal genomes using an ab initio algorithm with unsupervised training. Genome Res. 18:1979–1990. 10.1101/gr.081612.108. 18757608 PMC2593577

[jkaf256-B86] Wang H et al 2023. Uptake of oomycete RXLR effectors into host cells by clathrin-mediated endocytosis. Plant Cell. 35:2504–2526. 10.1093/plcell/koad069. 36911990 PMC10291037

[jkaf256-B87] Wang Q et al 2011. Transcriptional programming and functional interactions within the *Phytophthora sojae* RXLR effector repertoire. Plant Cell. 23:2064–2086. 10.1105/tpc.111.086082. 21653195 PMC3160037

[jkaf256-B88] Wang Y et al 2012. MCScanX: a toolkit for detection and evolutionary analysis of gene synteny and collinearity. Nucleic Acids Res. 40:e49. 10.1093/nar/gkr1293. 22217600 PMC3326336

[jkaf256-B89] Wang Y, Wang Y. 2018. *Phytophthora sojae* effectors orchestrate warfare with host immunity. Curr Opin Microbiol. 46:7–13. 10.1016/j.mib.2018.01.008. 29454192

[jkaf256-B90] Wawra S et al 2012. Secretion, delivery and function of oomycete effector proteins. Curr Opin Microbiol. 15:685–691. 10.1016/j.mib.2012.10.008. 23177095

[jkaf256-B91] Wheeler TJ, Eddy SR. 2013. Nhmmer: DNA homology search with profile HMMs. Bioinformatics. 29:2487–2489. 10.1093/bioinformatics/btt403. 23842809 PMC3777106

[jkaf256-B92] Whisson SC et al 2007. A translocation signal for delivery of oomycete effector proteins into host plant cells. Nature. 450:115–118. 10.1038/nature06203. 17914356

[jkaf256-B93] Wicker T et al 2007. A unified classification system for eukaryotic transposable elements. Nat Rev Genet. 8:973–982. 10.1038/nrg2165. 17984973

[jkaf256-B94] Xu S et al 2022. *Ggtree*: a serialized data object for visualization of a phylogenetic tree and annotation data. iMeta. 1:e56. 10.1002/imt2.56. 38867905 PMC10989815

[jkaf256-B95] Zhang Z et al 2024. Complete telomere-to-telomere genomes uncover virulence evolution conferred by chromosome fusion in oomycete plant pathogens. Nat Commun. 15:4624. 10.1038/s41467-024-49061-y. 38816389 PMC11139960

[jkaf256-B96] Zheng J et al 2023. dbCAN3: automated carbohydrate-active enzyme and substrate annotation. Nucleic Acids Res. 51:W115–W121. 10.1093/nar/gkad328. 37125649 PMC10320055

[jkaf256-B97] Zhu T et al 2025. Parabricks: GPU Accelerated Universal Pan-Instrument Genomics Analysis Software Suite. bioRxiv 666378. 10.1101/2025.07.23.666378, 27 July 2025, preprint: not peer reviewed.

[jkaf256-B98] Zion Market Research . 2023. Zion market research. [updated 2025 Apr 14]. https://www.zionmarketresearch.com/report/chocolate-market.

